# Comprehensive Analysis of Neonatal *versus* Adult Unilateral Decortication in a Mouse Model Using Behavioral, Neuroanatomical, and DNA Microarray Approaches

**DOI:** 10.3390/ijms151222492

**Published:** 2014-12-05

**Authors:** Akira Yoshikawa, Tomoya Nakamachi, Junko Shibato, Randeep Rakwal, Seiji Shioda

**Affiliations:** 1 Department of Anatomy, Showa University School of Medicine, 1-5-8 Hatanodai, Shinagawa, Tokyo 142-8555, Japan; E-Mails: yoshi-aki@med.showa-u.ac.jp (A.Y.); nakamachi@med.showa-u.ac.jp (T.N.); rjunko@nifty.com (J.S.); 2 Department of Physical Therapy, Faculty of Health Science, Tokoha University, 1-30 Mizuochicho, Aoi, Shizuoka 420-0831, Japan; 3 Laboratory of Regulatory Biology, Graduate School of Science and Engineering, University of Toyama, Toyama 930-8555, Japan; 4 Laboratory of Exercise Biochemistry and Neuroendocrinology, Institute of Health and Sports Sciences, University of Tsukuba, 1-1-1 Tennoudai, Tsukuba 305-8574, Japan; 5 Organization for Educational Initiatives, University of Tsukuba, 1-1-1 Tennoudai, Tsukuba 305-8577, Japan

**Keywords:** neonatal, adult, brain, motor functional recovery, DNA microarray, 8 × 60 K, transcriptome profiling, gene inventory

## Abstract

Previously, studying the development, especially of corticospinal neurons, it was concluded that the main compensatory mechanism after unilateral brain injury in rat at the neonatal stage was due in part to non-lesioned ipsilateral corticospinal neurons that escaped selection by axonal elimination or neuronal apoptosis. However, previous results suggesting compensatory mechanism in neonate brain were not correlated with high functional recovery. Therefore, what is the difference among neonate and adult in the context of functional recovery and potential mechanism(s) therein? Here, we utilized a brain unilateral decortication mouse model and compared motor functional recovery mechanism post-neonatal brain hemisuction (NBH) with adult brain hemisuction (ABH). Three analyses were performed: (1) Quantitative behavioral analysis of forelimb movements using ladder walking test; (2) neuroanatomical retrograde tracing analysis of unlesioned side corticospinal neurons; and (3) differential global gene expressions profiling in unlesioned-side neocortex (rostral from bregma) in NBH and ABH on a 8 × 60 K mouse whole genome Agilent DNA chip. Behavioral data confirmed higher recovery ability in NBH over ABH is related to non-lesional frontal neocortex including rostral caudal forelimb area. A first inventory of differentially expressed genes genome-wide in the NBH and ABH mouse model is provided as a resource for the scientific community.

## 1. Introduction

Clinical neurologists have long known that children who sustain brain damage during the neonatal period or early infancy have a greater capacity for motor function recovery than do adults. First, what does the literature tell us of age differences and brain injury? Let us look at some examples on cerebral hemispherectomy, which is one of the successful surgical procedures used in the treatment of pharmacologically untreatable epilepsy. Young children receiving a hemispherectomy do not have impaired motor performance post-surgery; moreover, they exhibit normal motor ability or improvement in the contralesional extremities [1,2,3,4,5]. In the case when such a surgery is performed between middle childhood and adulthood it is found that the patients become hemiplegic [6,7,8]. Similarly in rodents, it has been shown that adult animals with neonatal cortical lesion exhibit fairly skilled motor function in the contralesional forelimbs and hindlimbs. However, this is in marked contrast to the poor functional recovery of animals whose sensorimotor cortex (SMC) was lesioned between adolescence to adulthood [9,10]. All these studies point toward an age-dependent plasticity.

For the recovery of motor function after brain injury, corticofugal axonal projection especially corticospinal tract (CST) plays an important role. In rodents, the corticospinal tract originating from pyramidal neurons located in layer 5 of the SMC is developing after birth and it achieves topographic organization at least three weeks after birth [11,12,13,14]. The developmental CST and corticospinal neurons show a transient nature and are eliminated or became apoptotic. The CST axon transiently increases around postnatal first-week followed by a gradual decrease, and where axon projection is stable at or after postnatal three-weeks [15,16]. The corticospinal neurons are also transiently present [17]. During the first postnatal week, the neurons projecting to the medullary pyramid are distributed virtually throughout the entire cerebral cortex. In contrast, the distribution of such neurons is almost completely restricted to the SMC during adulthood [18]. Electrophysiological studies have also indicated that corticospinal axon temporarily project over all gray matter in the spinal cord, but superfluous axons existing in the ventral gray matter are eliminated with growth [19]. This axonal elimination has been demonstrated by slice co-culture [20]. Although not much has been done on the molecular aspects of brain injury in context of motor function, molecular mechanisms of axon guidance in the developing CST have been reported. One study reviewed the gene expression changes in the developing cortex [21], and the other looked at the pre-frontal cortex gene expression change using DNA microarray analysis [22].

The main compensatory function recovery mechanism in animals that underwent the CST injury, *i.e.*, stroke or spinal cord injury is that the unlesioned CST sends collateral sprouting fibers toward the lack of innervation side [23]. In neonatally hemidecorticated rats during development, the projection patterns from the undamaged SMC have been examined using anterograde labeling of the axonal fibers of pyramidal neurons. Pyramidal neurons project aberrant collateral fibers toward the contralateral red nucleus, contralateral superior colliculus, contralateral pontine nuclei, ipsilateral dorsal column nucleus, and ipsilateral gray matter of the cervical spinal cord [24]. Electrophysiological studies have indicated that the aberrant projections from the undamaged SMC side corticospinal tract mediate the lack of innervation extremities [25,26]. From these previous studies, we can say that the collateral sprouting plays a very important role in the compensatory mechanism after neonatal and adult brain hemisuction. Taking into consideration all of the above, we ask a question. Why is the motor function recovery capability different despite following the same compensatory mechanism?

Therefore to address the above question, we had been studying the development especially of corticospinal neurons using a rat model in conjunction with retrograde tracing studies. It was concluded that the main compensatory mechanism after unilateral brain injury at the neonatal stage was due to in part by non-lesioned ipsilateral corticospinal neurons that escaped selection by axonal elimination or neuronal apoptosis; a secondary temporary compensatory mechanism was also formed by sprouting collaterals [27]. However, the above result suggesting compensatory mechanism in neonate brain could not be correlated with high functional recovery at that time. Moreover, it raised another question, what is the difference among neonate and adult in context of functional recovery and mechanism(s) therein, especially focused on the corticospinal neurons? To the best of our knowledge there is no such study comparing the above.

In the present study, utilizing a brain unilateral decortication mouse model, we have compared motor functional recovery mechanism post-neonatal brain hemisuction (hereafter termed, NBH) with adult brain hemisuction (hereafter termed, ABH). In the first part of this ongoing research with an ultimate goal to unravel the mechanism(s), we performed three analyses: (1) Quantitative behavioral analysis of the forelimb movements using the ladder walking test, where the corticospinal system has been suggested to play an important role [28,29]; (2) neuroanatomical retrograde tracing analysis of unlesioned side corticospinal neurons; and (3) differential gene expression profiling in the unlesioned-side neocortex (rostral from bregma) in NBH and ABH using a high-throughput DNA microarray approach (8 × 60 DNA chip). The novelty of this study is the comparison between NBH and ABH for the compensatory and specifically corticospinal neurons related to the motor functional recovery in non-lesion neocortex by utilizing the classical retrograde tracing method. Thereafter, we have performed global genome analysis to unravel potential molecular factors/candidates related to the different ability of the motor functional recovery in both groups, a first such study utilizing the high-throughput DNA microarray approach. Moreover, the obtained data deposited at the NCBI, Gene Expression Omnibus (GEO) site (under accession number GSE59362) is freely available to the scientific community for download and further in-depth analysis.

## 2. Results

### 2.1. Cortical Hemisuction

**Figure 1:**
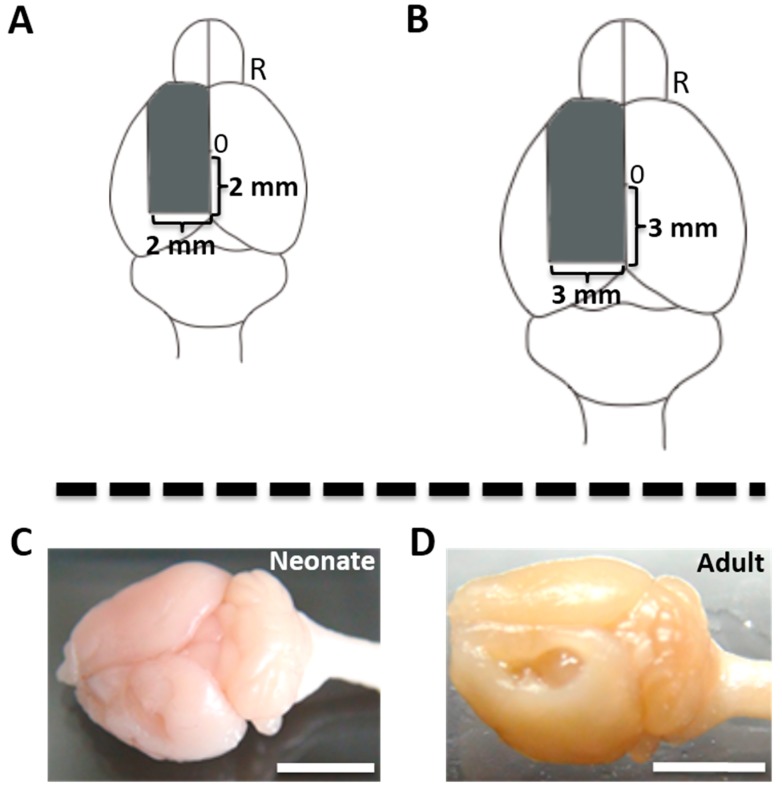

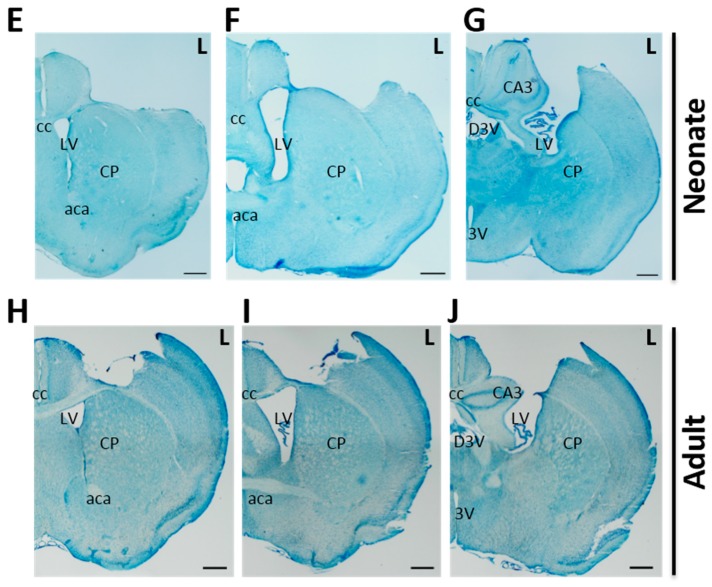
Overview of the surgery. Mouse that underwent left cortical hemisuction at P7 (**A**) and at 8–12 weeks (**B**). Gray area means aspiration area; (**C**,**D**) means the left cerebral cortex including the sensorimotor cortex (SMC) received a suction. All the figured photographs were taken at postoperative 3 months; Photomicrograph of the left side of coronal section in the 1 mm rostral from bregma (**E**,**H**), bregma level (**F**,**I**), 1 mm caudal from bregma (**G**,**J**) of a hemidecorticated mouse by Nissl staining; Neonatal brain hemisuction (NBH) group are (**C**,**E**–**G**); Adult brain hemisuction (ABH) group are (**D**,**H**–**J**). 0 = bregma point; R = right side; L = left side; cc = corpus callosum; LV = Lateral ventricle; CP = caudoputamen; aca = anterior commissure; CA3 = CA3 field, hippocampus; 3V = 3rd ventricle; D3V = dorsal 3rd ventricle; Scale bar = 5 mm (**C**,**D**) and 500 µm (**E**–**J**).

To generate left unilateral decortication model, the area including SMC was aspirated (Figure 1A,B). The mouse who received cortical hemisuction at P7 and adult stage, showed extensive left cerebral cortex covered with the SMC lesions (Figure 1C–J).

**Figure 2:**
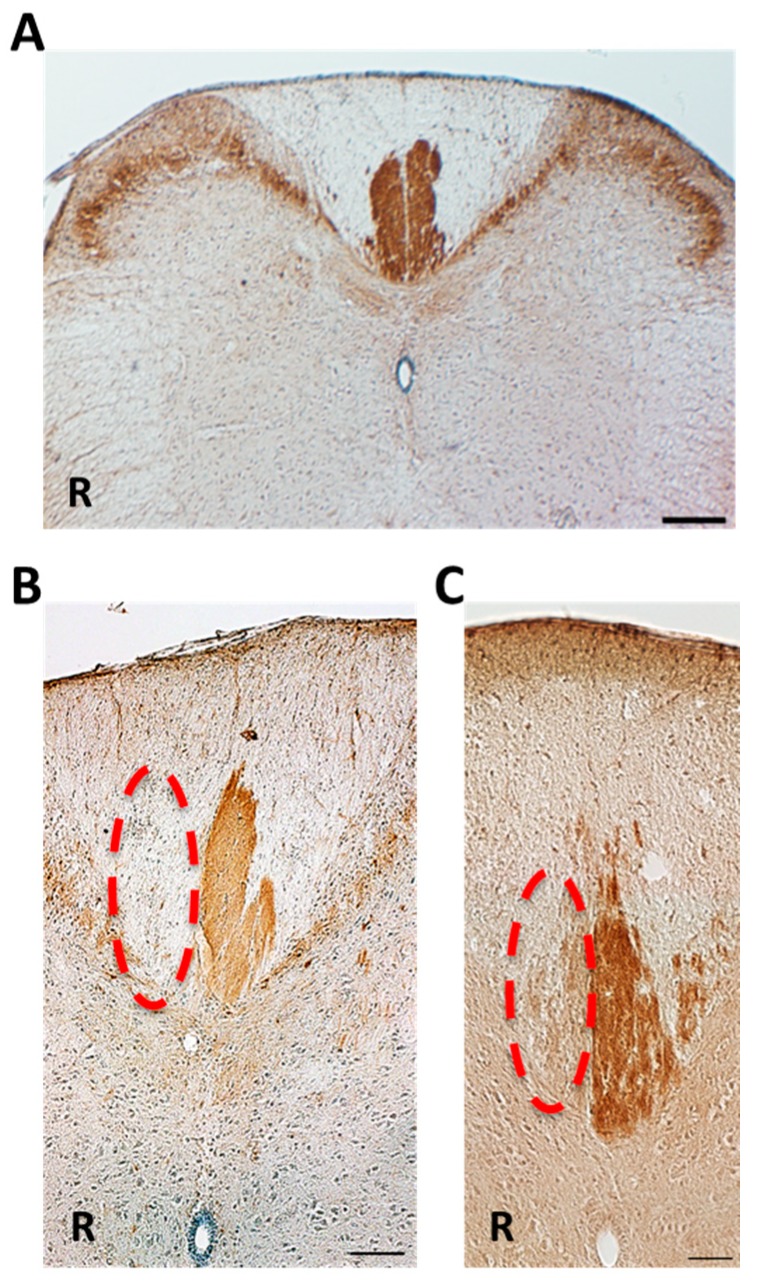
Protein kinase C-γ (PKCγ) immunohistochemistry. (**A**) Normal adult mouse. The corticospinal tract on both side were labeled with PKCγ antibody. On the other hand, an extensive absence of the left corticospinal tract was shown in an after postoperative 12 weeks’ mouse (dotted line); (**B**) NBH group; and (**C**) ABH group. R = right side; Scale bar = 200 μm (**A**) and =100 μm (**B**,**C**).

Next, we performed protein kinase C-γ (PKCγ) immunohistochemistry to identify the CST (Figure 2A). It was seen that the dorsal funiculus of CST almost disappeared in the right dorsal CST in both NBH and ABH (Figure 2B,C).

### 2.2. Ladder Rung Walking Test Reveals Difference of Motor Functional Recovery between NBH and ABH

**Figure 3:**
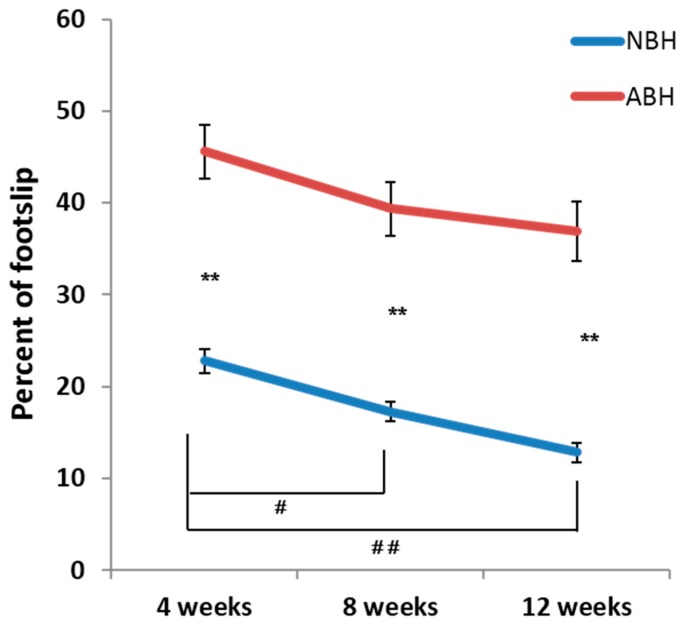
Horizontal ladder rung walking test reveals decreased percent of footslip in the NBH (*n* = 20) compared to the ABH (*n* = 12). In addition, there is sequentially significant difference on the number of footslips and percent of footslip in NBH (** *p* < 0.01, Mann-Whitney *U* test, ## *p* < 0.01 # *p* < 0.05, Kruskal-Wallis with the Tukey *post hoc* test).

The ladder rung walk test was conducted to assess skilled walking by quantifying the number of footslips, which evaluates digit, wrist, and forelimb motion recorded via a video camera (NBH: *n* = 20, ABH: *n* = 12, control: *n* = 9) (see, Supplementary Figure S1). The number of faulty placements of the right forepaw was counted during walking on a ladder rung at 4, 8 and 12 weeks after the injury. In control animals, the number of steps is 20 ± 1.2, the number of footslips is 1.1 ± 0.2 and percent of footslip per total steps is 5.5% ± 0.6% (data not shown). Although no significant differences could be observed on the number of steps between NBH and ABH in all evaluated points, there are sequentially significant differences in the number of steps in the NBH (Supplementary Figure S2A). A lower number of footslips and percent of footslip was observed in the NBH compared to ABH. In addition, there are sequentially significant differences in the number of footslips and percent of footslip in the NBH. Statistical analysis indicated a significantly greater recovery in the NBH at all phases (Figure 3).

### 2.3. Differences in Retrogradely Fluorogold (FG) Labeled Corticospinal Neurons in Unlesioned Cerebral Cortex

The FG was injected into the right side of lower cervical spinal gray matter diffusing from dorsal to ventral horn (Supplementary Figure S3A–C). There are three components of CST in the spinal cord-dorsal funiculus, dorso-lateral funiculus, and ventral funiculus, where axons terminate in the dorsal horn and the intermediate zone. Retrogradely FG labeled neurons are from these three components though we could not separate them among the three components.

**Figure 4:**
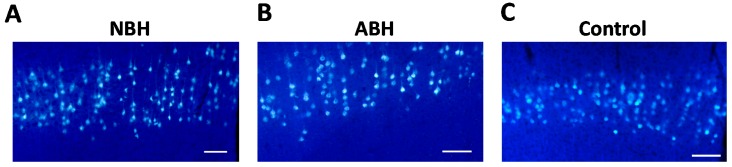

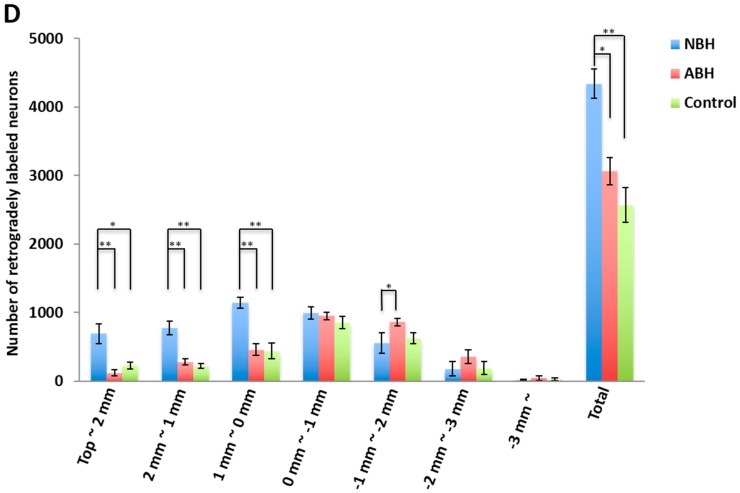
Retrograde tracing study. The corticospinal neurons in the right cerebral cortex were retrogradely labeled by FG in the 1 mm from bregma (**A**: NBH; **B**: ABH; **C**: control). The number of FG labeled neurons in the right cerebral cortex (**D**). Top means frontal pole and 0 mm means bregma level. The scale bar = 100 µm (**A**–**C**). ** *p* < 0.01, * *p* < 0.05, Kruskal-Wallis test followed by Steel-Dwass method.

To check whether the injection was carried out appropriately in each animal, we probed the injection site used by Image J. The area of injection site and the region of diffusion FG, and percent of diffusion region of FG were measured and calculated to normalize to the retrograde tracing method. There are no significant differences among these three components within the three groups (Supplementary Figure S3D–F). Results revealed that each animal was appropriately injected in a similar manner. The FG was injected into the right lower cervical level especially C5–C6 grey matter to confirm that the CST axon was targeted toward the ipsilateral side of cervical spinal cord (NBH: *n* = 7, ABH: *n* = 9, control: *n* = 8). The FG-labeled neurons in the unlesioned cerebral cortex may be related to the control of motor functional recovery in the right forelimb movement, one week after injection. Significantly more FG labeled neurons was observed in the NBH group than in the ABH and control groups of the rostral from bregma (Figure 4A–C). This area includes part of caudal forelimb area (CFA) and entire the rostral caudal forelimb area (RFA) [30,31,32]. Moreover, significant differences were noted in the total number of retrogradely FG labeled neurons in the unlesioned cerebral cortex (Figure 4D).

The above results on behavior and histological analyses indicated that (1) neonatal motor functional recovery was enhanced over adult; and (2) that the localization of corticospinal neurons in the non-damaged hemisphere, especially the rostral from bregma area was higher in NBH. These two findings suggested different ability of neonatal *versus* adult brain, and provided confidence to our decision to move to the next step of genome-wide transcriptome profiling to help unravel the underlying molecular mechanism(s) therein.

### 2.4. Total RNA Extraction, cDNA Synthesis, RT-PCR, and Experimental Strategy for DNA Microarray Analysis

**Figure 5:**
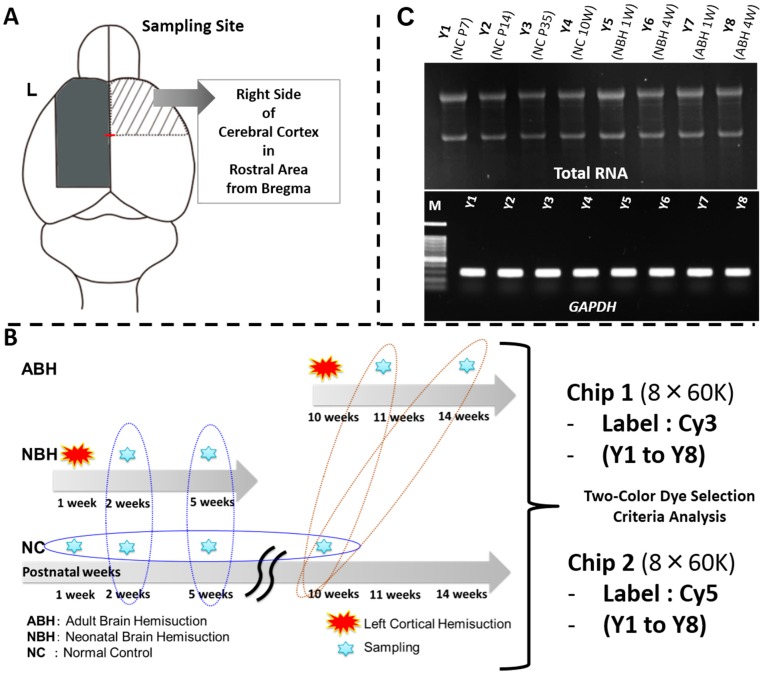
DNA microarray analysis of the brain region. (**A**) Sampling site of mouse whole brain and cerebral cortex sampled; (**B**) Sampling points and DNA microarray analysis on two 8 × 60 K microarray chips using a two-color approach. Brain tissues were ground to a fine powder in liquid nitrogen and stored at −80 °C; and (**C**) Total RNA extraction from the finely powdered brain tissues. Total RNA quality was confirmed by agarose-gel electrophoresis. Gel images for the *GAPDH* gene expression as a positive control by RT-PCR, and the PCR product bands stained with ethidium bromide. For further details, see the Experimental Section.

For total RNA extraction, the right side neocortex (rostral from bregma) from control, NBH and ABH at respective time points were prepared from dissected out samples as described in the Experimental section (Figure 5A,B). Good quality (A_260/280_ > 1.8; A_260/230_ > 1.8) total RNA was obtained in optimum quantity (>300 ng/µL) and used for cDNA synthesis, followed by semi-quantitative RT-PCR for checking mRNA expression of glyceraldehyde 3-phosphate dehydrogenase (*GAPDH*) as a positive control, in these eight samples (Figure 5C). It must be emphasized that the quality of the extracted total RNA is an extremely important step toward obtaining accurate mRNA expression profiling data at the whole genome level, in particular. After confirming stable and unchanged expression levels of *GAPDH*, and thus quality of the synthesized cDNA, we proceeded to design the microarray experiment. Two oligo DNA chips (8 × 60 K) were used for the present study, labeling each sample (Y1 to Y8) with either Cy3 and Cy5 for loading onto individual chips (Figure 5B). By this approach, we could obtain the gene expression data for each sample with both the Cy dyes, and that were therein utilized for downstream analyses. The gene expression data (genes differentially expressed in the NBH and ABH animals over the respective controls) are available as excel file worksheets under the series number GSE59362 at the NCBI Gene Expression Omnibus (GEO) public functional genomics data repository.

### 2.5. DNA Microarray Analysis Reveals Predominantly Down-Regulated Differentially Expressed Genes in NBH and ABH

**Figure 6:**
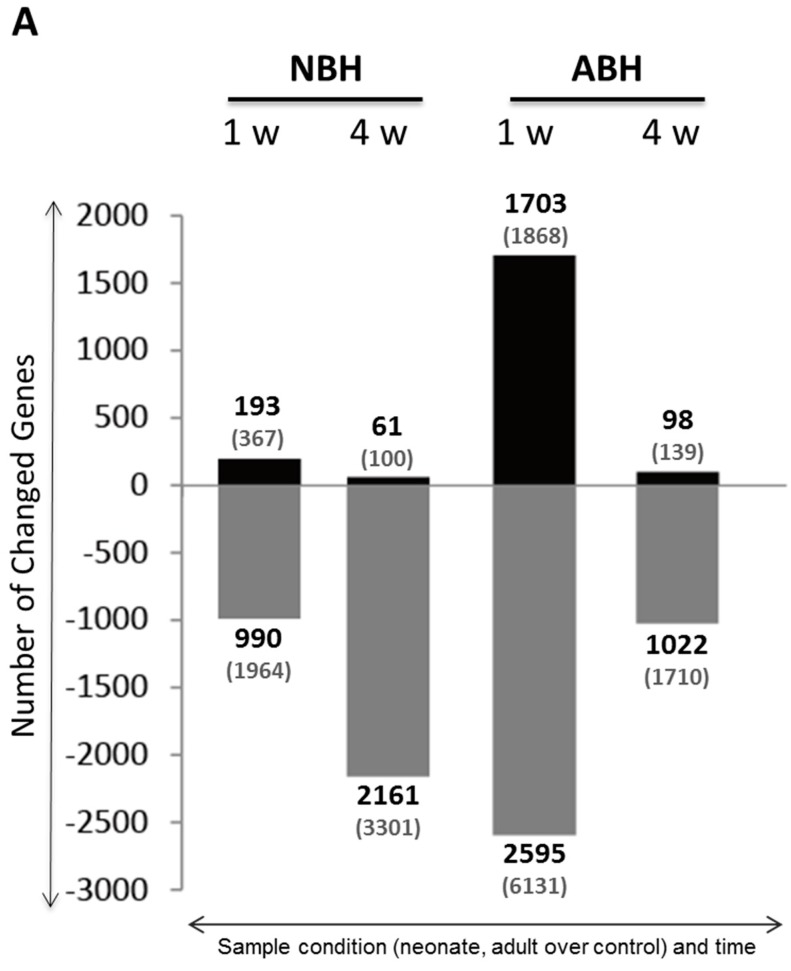

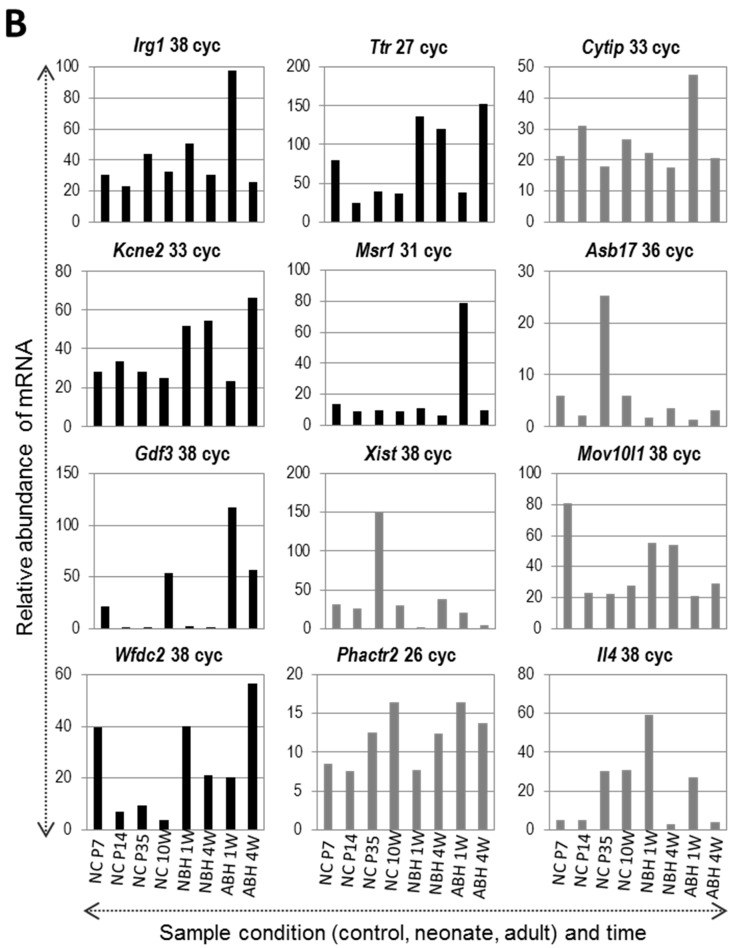
Differentially expressed genes in NBH and ABH, and validatory RT-PCR. (**A**) Graphs show the up- and down-regulated genes; and (**B**) Total RNA quality was confirmed by agarose-gel electrophoresis, and RT-PCR data (relative mRNA abundance) for randomly selected genes is shown. For further details, see the Experimental Section.

Results of the transcriptome profiling data are summarized in Figure 6. In the NBH animals, a total of 193 and 61 genes were up-regulated while 990 and 2161 genes were down-regulated at 1 week and 4 weeks, respectively. On the other hand, in the ABH animals, a comparatively larger number of genes (1703 up-regulated and 2595 down-regulated) were changed at 1 week over 98 and 1022 up- and down-regulated genes, at 4 weeks (Figure 6A). It should be noted that these gene numbers are those that have been identified and named; the total number of up- and down-regulated genes are given in parenthesis in the graph presented in Figure 6A. These results reveal one characteristic of the gene expression changes, namely down-regulated gene expression predominates these two developmental stages with cortical hemisuction. Interestingly, in the ABH but not NBH animals, the nearly two-fold increased down-regulations were observed earlier, *i.e.*, at 1 week after injury.

### 2.6. Confirmatory RT-PCR Analysis of Gene Expression Changes after DNA Microarray

Semi-quantitative RT-PCR analysis data on selected up- and down-regulated genes (Supplementary Tables S1–S4: NBH animals, 1 week up- and down-, and 4 week up- and down-regulated; Supplementary Tables S5–S8: ABH animals, 1 week up- and down-, and 4 week up- and down-regulated) validated the DNA microarray data, *i.e.*, induced genes were found to have increased expression (graphs with black colored bars) and suppressed genes were found to have decreased expression (graphs with grey colored bars). By carrying out the RT-PCR analysis of all samples with specifically designed primers, as described in the Experimental Section and detailed in Table 1, we could also observe the mRNA expression level of each gene in each of the developmental stages analyzed in this study (Figure 6B). For example, the *Ttr* (transthyretin) gene, encoding for a blood and cerebrospinal fluid (CSF) protein that has neuroprotective effects [33,34], was strongly induced at 1 week NBH and showing only a slight decline in expression at 4 weeks. On the contrary, *Ttr* expression was only strongly enhanced at 4 weeks ABH. These data also showed good correlation with the obtained gene expressions obtained using the microarray analysis (Supplementary Tables S1, S3 and S7). Among the down-regulated genes, Xist [35] showed a very low level of expression at 1 week in the NBH, and at 4 weeks in ABH (Figure 6B), which matched well with the microarray data (Supplementary Table S4).

### 2.7. Functional Categorization of Differentially Expressed Genes Reveals Up-Expression and Down-Expression of Diverse Functions

Functional categorization of genes revealed that at both 1 and 4 weeks after injury, the NBH animals showed down-regulation as the predominant functions, with Hedgehog signaling pathway, neurogenesis, neural stem cells and G protein coupled receptors as the first 3 abundantly influenced categories at 1 week compared to epidermal growth factor/platelet-derived growth factor (EGF/PDGF) signaling as the main category at 4 weeks (Supplementary Tables S10 and S12). The up-regulated genes showed abundance of expression in atherosclerosis, inflammatory response and autoimmunity, and NF-κB signaling pathway categories at 1 week compared to NF-κB signaling pathway as the main category at 4 weeks (Supplementary Tables S9 and S11). On the other hand, in the ABH animals, at 1 week an opposite effect was seen, with up-regulated genes abundantly expressed in categories of osteogenesis, epithelial to mesenchymal transition and innate and adaptive immune response (Supplementary Table S13). However at 4 weeks, the down-regulated genes were predominant in categories of EGF/PDGF signaling, G protein coupled receptors and molecular toxicology pathway finder (Supplementary Table S16). The gene functions for all the NBH and ABH animals at 1 and 4 weeks post-hemisuction are listed in Supplementary Tables S9–S16.

## 3. Discussion

### 3.1. Behavior and Histological Analyses of Corticospinal Neurons in Non-Lesioned Hemisphere

Here, we compared motor functional and morphological recovery in NBH *versus* ABH animals that underwent the left cortical hemisuction, in regards to compensatory descending spinal projections from non-lesional cerebral cortex to the right cervical spinal cord. Our results confirmed the higher recovery ability in NBH over ABH is related to non-lesional frontal neocortex including RFA.

**Table 1:** Primer design for RT-PCR validation experiment. Primers are designed in-house and original to our group.

Accession (Gene)	Gene Symbol	Description	Nucleotide Sequence (5'–3'): Forward *	Nucleotide Sequence (5'–3'): Reverse *	Product Size (bp)
NM_008084	*Gapdh*	glyceraldehyde-3-phosphate dehydrogenase	gctacactgaggaccaggttgt	ctcctgttattatgggggtctg	306
NM_008392	*Irg1*	immunoresponsive gene 1	accttctatggtcactggagga	tgcaacgtcgtttagatattgg	266
NM_013697	*Ttr*	transthyretin	caccaaatcgtactggaagaca	ataagaatgcttcacggcatct	285
NM_139200	*Cytip*	cytohesin 1 interacting protein	gagtcgtccttgtttggaaatc	aagggagaaaaggacccactac	290
NM_134110	*Kcne2*	potassium voltage-gated channel, Isk-related subfamily, gene 2	tacgtcatcctgtacctcatgg	tgtcttctgagcaagcacaagt	314
NR_001463	*Xist*	X-inactive specific transcript	aaggtggcttgctatggtaaaa	ggaactgcattaaagtcccaac	275
NM_025758	*Asb17*	ankyrin repeat and SOCS box-containing 17	tacgtggctcagacaagacagt	tgatggttattctgcaaaggtg	337
NM_008108	*Gdf3*	growth differentiation factor 3	aagaacgtcattctccgacatt	acaagaagcccagctactatgc	264
NM_031195	*Msr1*	macrophage scavenger receptor 1	cgtgaatctacagcaaagcaac	gtaagccctctgtctccctttt	257
NM_031260	*Mov10l1*	Moloney leukemia virus 10-like 1	acataaaggttggctcggtaga	ccctgtgtagacaccattgcta	260
NM_026323	*Wfdc2*	WAP four-disulfide core domain 2	ctctgtctgctccaagcctaat	catttcatgttgccagaacact	276
NM_001195065	*Phactr2*	phosphatase and actin regulator 2	ccaacaacggaagaattagagc	tcatgaacttccatttctgtgc	296
NM_021283	*Il4*	interleukin 4	cctgctcttctttctcgaatgt	tttcagtgatgtggacttggac	346

* Primers are designed in-house, and original to our laboratory.

In this study, we first carried out a quantitative behavioral analysis of the right forelimb movements using the ladder rung walking test. This test is needed to find the ability of fine movement in forelimb, and also suggested that the cortical motor control system plays a major role in this test [28,29]. From this behavioral analysis, a lower number of footslips and percent of footslip was observed in the NBH compared to ABH. In addition, there are sequentially significant differences in the number of footslips and percent of footslip in the NBH. These results suggest the greater recovery in the NBH at all phases. From this assesment, we concluded that the motor functional recovery of the right forelimb in animals that underwent the left cortical hemisuction as neonate was more considerably restored than in adult animals. Some previous studies have also supported the fact that the animals receiving cortical lesions during the immature stage more clearly recovered and had less motor disfunction than mature animals [9,10,11,12,13]. These motor functional recovery mechanisms after damage to the corticospinal system have been suggested to involve reorganization of neural circuit, especially focused on non-lesional corticospinal tract [24,25,27,30,32,36,37].

However, these studies have investigated NBH and ABH as different models and protocols. Therefore, we performed retrograde tracing study after the behavioral analysis and confirmed the different phenomenon in both groups. In the present study, retrogradely labeled corticospinal neurons projecting to the ipsilateral C5–C6 spinal gray matter were found to be more significantly different in the motor region mainly and entirely on the contralesional neocortex in the NBH over ABH animals. Notably, it was confirmed that more ipsilateral corticospinal projecting neurons were located in the contralesional rostral area from bregma in the NBH than ABH animals. Previous study has indicated that contralesional cortex was controlling the movement of both forelimbs in the rats receiving unilateral decortication in neonate, moreover the corticofugal axons from contralesional motor cortex area projected into the ipsilateral spinal gray matter [24]. The same research group subsequently indicated that these areas were part of the caudal forelimb area (CFA) and the rostral caudal forelimb area (RFA) [32]. Because our retrogradely labeld neurons were shown to be present in the rostral region from bregma, it is our belief that the retrogradely labeled neurons are related to the neurons belonging to the region of CFA and RFA, respectively, and might play an important role in providing more compensatory motor functional recovery in NBH than ABH. It is believed that the reorganization within the CFA and RFA after central nervous system lesion probably undergoes different compensatory processes [30,32,38]. As a future study, we are planning to conduct a detailed brain mapping of the region bordered by CFA, RFA. Moreover, we want to know the different projections from CFA and RFA to the spinal gray matter by using anterograde tracing method.

Taken together, comparing the ability in a single study might be meaningful to understand the underlying phenomena why young (infant) of animals or children can regain the neurological function but is not sufficient for understanding the underlying mechanism(s). Therefore, we performed a global analysis to screen out the underlying molecular factors, in this case, genes that might be related to the neuroprotection factors, neurogenesis factors, neurogrowth factors or collateral sprouting factors, escape of axonal elimination and neuronal apoptosis factors *etc.*, on the contralesional neocortex, rostral from bregma in which retrogradely labeled neurons were observed more in the NBH than ABH animals. These gene analysis data are discussed further below.

### 3.2. Genome-Wide Gene Expression Analysis of NBH versus ABH Mouse

The study presented here provides new insight into the differentailly expressed genes in the NBH and ABH animals, and serves as a important resource for the scientific community. Several genes identified here are novel in context to their function/role in unilateral decortication, therefore, these gene inventories from mouse brain at both young and adult stages post-cortical hemisuction may have important but unknown roles in the compensatory mechanims being discussed. We discuss briefly some genes and gene categories, first, the top up- and down-regulated genes selected for RT-PCR analysis, and second, the categories and some gene functions therein.

Looking first among the 12 selected genes for RT-PCR experiments, we found the strongest upexpression of *Ttr* (transthyretin) gene in both the NBH (1 and 4 weeks) and ABH (4 weeks) animals over the controls (Figure 6B). As reported by Santos and coworkers, their research on the brain of TTR null mouse showed increased vulnerability to ischemic damage [33]. Moroever, those authors further suggested that the CSF-source TTR was important to neuroprotection. Our data on higher expression levls of *Ttr* gene in NBH, early on, rather than ABH, later on, suggest that the TTR might not only be involved in neuroprotection but also that it has a greater role in the neonates based on the observed high early transcription response at 1 week. Interestingly, a gene known to be induced under inflammatory conditions, namely *Irg1* (immunoresponsive gene 1), was strongly upexpressed especially in ABH 1 week (Figure 6B). *Irg1* is also one of the most highly upexpressed genes under proinflammatory conditions such as bacterial infections [39]. Though the biological role of *Irg1* remains unknown, a paper by Michelucci and coworkers suggests that the gene has a function in the immune response, and was reported as an enzyme to produce antimicrobial metabolites [39]. The adult over young difference in upexpression of *Irg1* might be due to a higher degree of immunity or enhanced immune response required in the former to help the brain cells/tissues to recover from the injury/inflammation. What and how molecular factors are involved therein might form a separate and interesting investigation in the future. Among the downexpressed genes, *Xist* one of the first discovered long noncoding RNAs (lncRNAs) [40] is enigmatic as we have no idea of its possible role in the ischemic condition in this experiment. This lncRNA mediates X chromosome inactivation, a seminal developmental process particularly important in the brain [41]. However, a recent review on the ncRNAs seems to implicate the RNA-based networks in the molecular pathogenesis of stroke [42].

The gene functional category analysis further revealed clear differences among the NBH and ABH groups (Supplementary Figures S4 and S5). From these results, one can conclude that the recovery mechanism(s) post-cortical hemisuction involve both up- and down-regulation of genes but downexpression of genes is the predominant characteristic in NBH animals compared to predominatly upexpressed genes in ABH animals. Interestingly, downexpression of genes persisted even at 4 weeks. Taken together, the identification of numerous gene candidates in this study comparing neonatal with adult brain hemisuction via a microarray-based approach may help not only target individual genes for further study, but also serve to find the underlying mechanisms and answer biological questions behind high compensatory motor functional recovery mechanisms.

## 4. Conclusions

### 4.1. Clinical Issues

Early diagnosis and treatment of patients with brain injury is critical in order to avoid the effects of brain damage and thereby improve survival outcomes. Clinical neurologists have long known that children who sustain brain damage during the neonatal period or early infancy have a greater capacity for motor function recovery than do adults. As an example, hemispherectomy is surgery for the treatment of epilepsy performed for infants or children who have intractable seizures. This surgery removes part of abnormal cortex and/or subcortex. Resulting brain damage, stroke or trauma to motor area, generally results in paralysis of contralateral musculature and disruption of skilled limb use. However, children post-surgery often exhibited normal motor function or mild or slight paralysis. Why this is so remains unclear. Elucidation of the mechanism helps us, including those involved in the rehabilitation field, understand the brain’s ability for resistance and recovery from injury, and to aid prognosis prediction. Appropriate diagnosis and prognosis prediction is very important for effective rehabilitation of patients.

### 4.2. Results

Using the whole genome mouse 8 × 60 K DNA microarray system with the two-color approach, our results have provided a first inventory of the differential expressed gene expressions genome-wide in the NBH and ABH mouse model, a resource not only for us but also the scientific community, and on which basis further bioinformatics and functional analyses will be carried out in future studies. In ABH group, genes related to immune system (e.g., interleukins, macrophage, immunoglobulin, *et al*.) are up-regulated post 1-week brain injury, and regeneration and repair factors start to be upexpressed at 4 weeks after brain injury. In NBH group, genes related to immune system are up-regulated after 1 week, as well as neuroprotective factors (e.g., *Ttr*) and regeneration factors (e.g., folate receptor 1). In addition, apoptosis factor (e.g., caspase 14) was down-regulated. Especially, *Ttr* was highly interesting as its strong upexpression was seen at 1 week in NBH group but not in ABH group, where it was induced only at 4 weeks. Comparative and time-dependent analysis of the brains of hemidecorticated mouse provided an inventory of gene candidate’s upexpressed or downexpressed in sync with the onset of “brain injury”, and justified our investment in the genome-wide global gene expression analysis approach.

### 4.3. Implications and Future Directions

This study fulfils the study objectives by (1) providing researchers with a powerful resource in terms of gene candidates that are up- or down-regulated in the “brain injury”, e.g., hemispherectomy; and (2) allows us to differentiate early and late gene expressions that are involved not only in regulating brain injury but also in its progression. Our results also confirm the importance of this work in shedding light on mechanisms underlying brain injury. Two future works arising from this gene resource are as follows. First, it will be possible to analyze and study these up and predominantly down-regulated genes and explain how and why neonates show early recovery post-cortical hemiscution compared to the adults. The answers will be addressed in future studes using both deep bioinformaic analysis (Ingenuity Pathways Analysis, IPA; Ingenuity^®^ Systems, www.ingenuity.com). Second, gene candidates identified here could be targeted in experiments involving a knock-out mouse (e.g., *Il6* KO) for uncovering gene regulatory processes and elucidating their effects on brain injury. Some genes, such as *Cxcl*/*Ccl* for example, could also be used for specific experiments designed to test their potential in drug therapy. Finally, this study forms the basis for future research to examine the influence of selected neuropeptides to reverse the effects of brain injury and provide clues about the molecular mechanisms underlying this process. It is critical that we continue our investment in the global analysis of gene and subsequent protein expression in brain injury mouse models to unravel the underlying molecular basis. This study indicated many gene candidates that are potentially related to compensatory motor functional recovery mechanism in mouse that underwent the left cortical hemisuction at P7 and adult age. We hypothesize that the compensatory motor functional recovery mechanism occurring in the neonatal cortical hemisuction might be related to the collateral sprouting along with escaped selection by axonal elimination or neuronal apoptosis. Therefore, we focus on gene categories and factors related to nerve growth, neuroprotection, regulation of neuronal apoptosis, and regulation of regeneration and repair toward the central nervous system. Further molecular functional analysis, will ultimately help clarify the relation to the age-dependent compensatory motor functional recovery mechanisms and gene function.

## 5. Experimental Section

### 5.1. Animals and Husbandry

C57BL/6J mice were purchased from Japan Charles River (Kanagawa, Japan). The animals were housed at the Animal Institution in Showa University. Mice were maintained in cages in a ventilated animal room with controlled temperature and relative humidity with a 12 h light/12 h dark schedule (lights were turned on at 8:00 AM) and had access to laboratory chow and tap water *ad libitum*. Neonatal mice born from pregnant females were kept with the mother during the lactation period and weaned at postnatal four-weeks. All animal care and experimental procedures were approved by the Institutional Animal Care and Use Committee of Showa University (approval number, M6031).

### 5.2. Left Unilateral Decortication Model

The animals were deeply anesthetized with 2.5%–3.5% sevoflurane (Mylan Inc., Canonsburg, PA, USA) in a 30% O_2_ and 70% N_2_O gas mixture inhalation via a face mask, and fixed in a stereotaxic frame. They were subjected to cortical hemisuction at day 7 (NBH group) and 8–12 weeks (ABH group) after birth. In the NBH group, we decided to aspirate the area of the left cerebral cortex including the sensorimotor cortex (SMC) according to a previous study [18]. The aspiration area was caudal 2 mm and lateral 2 mm from bregma level toward the frontal pole (Figure 1A). In the ABH group, we aspirated area of left cerebral cortex including the SMC as per previous studies [31,43]. The aspiration area was caudal 3 mm and lateral 3 mm from bregma level toward the frontal pole (Figure 1B). Briefly, the cranial bone was carefully removed with a dental drill, exposing the cortical surface. Following retraction of the dura mater, the neocortical parenchyma was aspirated using a micropipette tip connected with an aspirator. After cortical hemisuction, the cranial bone was returned to the same position and the skin opening sutured with 7-0 silk (NBH) and 4-0 silk (ABH). The animals were kept in a recovery chamber (37 °C) for approximately 30 min for recovery from their anesthesia, and then returned to their home cages.

### 5.3. Behavioral Test

NBH (*n* = 20, male/female (M/F); 8/12) and ABH (*n* = 12, male/female (M/F); 5/7) were evaluated for motor function by ladder rung walking test 4, 8 and 12 weeks after cortical hemisuction to compare steps of developing animals because maturation of stepping is achieved at the 3rd to 4th week of age. Age-matched control animals (*n* = 9, male/female (M/F); 5/4) were also examined by the ladder rung walking test (Supplementary Figure S1A). The apparatus for ladder rung walking test consisted of two 82 cm × 15 cm clear acrylic plates having 1 cm interval small hole and approximately 1 mm diameter round bamboo bars. The plates were placed parallel and horizontally on 12 cm height stages with 10 cm distance, and each bamboo bar was inserted into the small hole with 2 cm interval. The distance between two stages were fixed to 70 cm and evaluation of the behavior was conducted with 60 cm distance among them (Supplementary Figure S1B,C). Briefly, the mice fasted overnight and were put on the ladder rungs (Supplementary Figure S1D). The training was conducted for approximately 10 min for each mouse until sufficient learning, and the walking of each mouse was recorded with video camera (FDR-CX700V Handycam; Sony, Tokyo, Japan) for five times. The video camera was positioned at a ventral angle in order to record clearly the right forelimb and paw positions, and data analysis was performed a frame-by-frame using the recorded document. The numbers of total and error (footslip) steps on right forelimb were counted and data was expressed percentage of footslip per total steps. Definition of the footslip is the right wrist joint falling under the bar as well as the right forelimb falling completely (Supplementary Figure S1E,F).

### 5.4. Retrograde Neuronal Tracing Study

The retrograde fluorescent tracer FG was purchased from Biotium Inc. (Hayward, CA, USA; Cat.: 80014). After completing the behavioral test at postoperative 12 weeks, the mouse (NBH = 7, ABH = 5, Control = 8) were anesthetized with 2.5%–3.5% sevoflurane in a 30% O_2_ and 70% N_2_O gas mixture (2:1) inhalation, and fixed in a stereotaxic frame. Laminectomy at vertebral levels C3–C7 was performed on mouse that had received neonatal and adult cortical hemisuction and on normal adult mouse. A retrograde tracer, FG (4%) was dissolved in sterile saline, and 1.0 µL of the solution was injected into the right hemisphere of C5–C6 spinal cord (at 0.6 ± 0.1 mm from midline and 1.0 mm depth) using glass syringes and needles (32GA RN; Hamilton, Reno, NV, USA) for 30 s by a micropump device (UltraMicroPump II and Micro4™, WPI Inc., Sarasota, FL, USA). The incisional wound was sutured with 4-0 silk, and the mice were kept in a 37 °C chamber until recovery from anesthesia. Seven days after injection, the mouse was transcardially perfused with saline containing 1% heparin and 4% paraformaldehyde (PFA) in a 0.1 M phosphate buffer (PB, pH 7.4) under sodium pentobarbital anesthesia. Brains and cervical spinal cord were removed and postfixed in 4% PFA overnight at 4 °C. After immersion in 20% sucrose (0.1 M PB) at 4 °C for cryoprotection, the brains and spinal cord were embedded in Tissue-Tek (Sakura Finetek, Tokyo, Japan), and frozen in liquid nitrogen. The frozen specimens were cut serially into 40 µm coronal sections in a cryostat (HYRAX C50, Carl Zeiss, Oberkochen, Germany) and thaw-mounted on MAS-coated glass slides (Matsunami, Osaka, Japan).

### 5.5. Staining

Three sets of serial sections were cut. One series was used to count retrogradely labeled neurons in the right neocortex. The other two series were used to evaluate the left cortex lesion and identify each nuclear profile. Cerebral slices were Nissl stained, and the cervical spinal cord slices were stained for PKCγ immunohistochemistry analysis as described below.

### 5.6. PKCγ Immunohistochemistry

To validate the left cortical hemisuction, cervical spinal cord was immunostained with PKCγ antibody. It has been reported that the corticospinal tract highly expressed PKCγ and the expression is known to decrease after cortical injury [29]. Briefly, sections from the cervical spinal cord were incubated in 0.3% hydrogen peroxide in methanol for 1 h to quench endogenous peroxidase activity, and then immersed for 1 h in 5% normal horse serum (NHS) to block a non-specific reaction. The sections were then incubated with rabbit antibody against PKCγ (C-19, 1:1000; Santa Cruz Biotechnology, Inc., Santa Cruz, CA, USA) in 5% NHS overnight at 4 °C. After rinse with PBS several times, the sections were incubated with biotinylated-conjugated goat anti-rabbit IgG (Cat.: 111-036-003, 1:200; Jackson ImmunoResearch, West Grove, PA, USA) for 2 h at room temperature. They were then incubated in an avidin-biotin complex solution (VECTASTAIN ABC kit; Vector Laboratories, Burlingame, CA, USA) followed by diaminobenzidine (Sigma–Aldrich, St. Louis, MO, USA) as a chromogen. The sections were dehydrated in series of alcohol and xylane, and enclosed by malinol (Cat.: 2009-2, Muto Pure Chemicals Co., Ltd., Tokyo, Japan).

### 5.7. Neuronal Counts

Prior to performing the retrogradely labeled neuron count, a check on the injection method was carried out by examining the injection site using the Image J free software version 1.46r (National Institutes of Health). First, the area of right cervical spinal cord was measured (A) followed by measuring the diffusion region of FG in that same area (B). We calculated the rate of diffusion as percent of 100% values in the injection site using the following formula: percent of diffusion = B/A. These calculations were performed on three sections per one mouse: center of the injection point, and rostral pint and caudal point from center of the injection point. These intervals between the measured slices were 80 µm. Next, we calculated the average value of these three points and calculated the average value per one group. After completing the above examination, we then counted the retrogradely labeled neurons in the right neocortex that sent axon terminals or collaterals to the C5–C6 spinal gray matter. For counting, we used one of the three prepared series of sections. The intervals between the counted slices were 80 µm, and therefore multiple counts of individual neurons were avoided. Only the neurons that were clearly fluorescent were used in the counting step. The number of labeled neurons in the right neocortex was counted and added to 1 mm interval separately. The area of the right neocortex was determined on the basis of Nissl stained sections referred to the mouse brain atlas [44].

**Figure 7:**
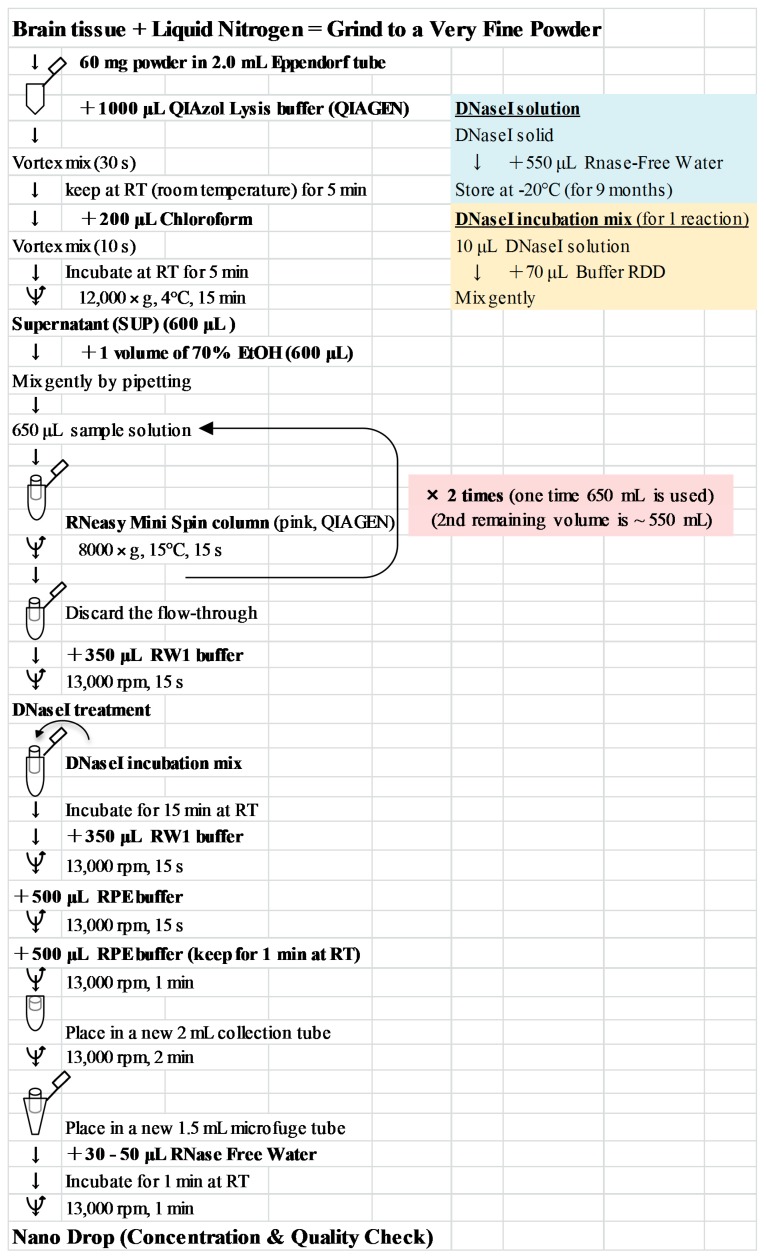
Illustrated protocol for extraction of total RNA from the brain tissue.

### 5.8. Total RNA Extraction, cDNA Synthesis, and RT-PCR

In order to confirm different motor functional mechanism between NBH and ABH, the right side neocortex (rostral from bregma) was individually ground to a very fine powder with liquid N_2_ as previously described [45,46]. Sample powders were stored at −80 °C till used for RNA or DNA extraction. Total RNA was extracted from 60 mg sample powder using the QIAGEN RNeasy Mini Kit (QIAGEN, Maryland, MD, USA) [45,46,47,48]. The protocol for total RNA extraction is provided in Figure 7. To verify the quality of this RNA, the yield and purity were determined spectrophotometrically with NanoDrop (Thermo Scientific, Wilmington, DE, USA) and confirmed using formaldehyde-agarose gel electrophoresis. cDNA synthesis and quality check by RT-PCR was performed as also described previously [45,46,47,48]. To check the quality of the synthesized cDNA, RT-PCR was performed to confirm the expression of the *GAPDH* gene (Figure 5 and Table 1).

### 5.9. Global Gene Expression Analysis

In order to confirm differential gene expression between NBH and ABH, the right side neocortex (rostral from bregma) was individually ground to a very fine powder with liquid N_2_ as mentioned above followed by genome-wide analysis. Briefly, sample powders were stored at −80 °C till used for RNA or DNA extraction. Following total RNA extraction, quality and quantity check, cDNA synthesis and RT-PCR, the sample was considered to be appropriate for use in the DNA microarray analysis. The reasons for multiple checks and use prior to DNA microarray analysis was first, due the high cost incurred during this analysis, and second, and most importantly, to obtain, as far as possible, accurate results. Gene expression changes can be easily misinterpreted in case any of the above steps are not followed precisely. Total RNA extracted from each control, NBH and ABH experiment was pooled in each group, prior to DNA microarray analysis (Agilent mouse whole genome 8 × 60 K; G4852A) performed essentially as described previously [45,46,47,48] with some modifications. We designated four male animals in each groups (Control, NBH and ABH) (Figure 5). We performed DNA microarray analysis using two slides composed of 8 chips each, labeling one set with Cy3 and the other with Cy5. We searched genes which are altered in the NBH and ABH compared to the control.

Briefly, total RNA (250 ng) was labeled with either Cy3 or Cy5 dye using an Agilent Low RNA Input Fluorescent Linear Amplification Kit (Agilent, Santa Clara, CA, USA). Fluorescently labeled targets of control as well as treatments were hybridized to the same microarray slide with 60-mer probes. A two-color labeling with Cy3 and Cy5 dyes was followed to nullify the dye bias associated with unequal incorporation of the two Cy dyes into cDNA. To select differentially expressed genes we considered genes that were up-regulated in chip 1 (Cy3 label for control and NBH/ABH, respectively) but down-regulated in chip 2 (Cy5 label for control and NBH/ABH, respectively). The use of a two-color approach provided a more stringent selection condition for changed gene expression profiling than use of a simple single-color approach [45,46,47,48]. Hybridization and wash processes were performed according to the manufacturer’s instructions, and hybridized microarrays were scanned using an Agilent Microarray scanner G2505C. For the detection of significantly differentially expressed genes between control and cortical hemisuction samples each slide image was processed by Agilent Feature Extraction software (version 11.0.1.1). This program measures Cy3 and Cy5 signal intensities of whole probes. Dye-bias tends to be signal intensity dependent therefore, the software selected probes using a set by rank consistency filter for dye-normalization. Said normalization was performed by LOWESS (locally weighted linear regression) which calculates the log ratio of dye-normalized Cy3- and Cy5-signals, as well as the final error of log ratio. The significance (*p*) value based on the propagate error and universal error models. In this analysis, the threshold of significant differentially expressed genes was <0.01 (for the confidence that the feature was not differentially expressed). In addition, erroneous data generated due to artifacts were eliminated before data analysis using the software.

The list of genes searched in the brain (neocortex) was further analyzed following our previous studies [45,46,47,48]. In this analysis, each gene was looked for its functional category, and subcategory. To re-check the microarray data semi-quantitative RT-PCR as detailed above was performed on interesting genes using gene specific primers (Table 1). Each gene expression analysis was performed at least twice as independent PCR reactions and electrophoresis on gel, and one of the images was presented as a representative data for each gene in the respective figures for up-regulated (≥1.5-fold) and down-regulated (≤0.75-fold) expressions.

### 5.10. Access to Gene Array Data

The outputs of microarray analysis performed in this study are available under the series number GSE59362 (http://www.ncbi.nlm.nih.gov/geo/query/acc.cgi?acc=GSE59362), at the NCBI GEO public functional genomics data repository (http://www.ncbi.nlm.nih.gov/geo/info/linking.html).

### 5.11. Functional Categorization

Pathway and disease states-focused gene classification of the differentially expressed genes in the neocortex of NBH and ABH animals were classified based on the available categories of more than 100 biological pathways or specific disease states in the SABiosciences PCR array list (QIAGEN; www.sabiosciences.com) for *Mus musculus*. The numbers in the *y*-axis represent the number of genes in each category, which are indicated on the *x*-axis.

### 5.12. Statistical Analysis

Behavioral tests were analyzed by Mann-Whitney U test to compare the percent of footslip in the NBH group with the ABH group at every week. In addition, we conducted statistical analysis so as to confirm the sequential change within each group using Kruskal-Wallis with the Tukey *post hoc* test. Fluorogold diffusion region and Fluorogold-labeled corticospinal neurons were analyzed by Kruskal-Wallis test following the Steel-Dwass method. All data were presented as mean ± SEM (standard error of the mean), and statistical significance was accepted at *p* < 0.05 and 0.01.

## Supplementary Materials

Supplementary materials can be found at http://www.mdpi.com/1422-0067/15/12/22492/s1.

## Abbreviations

ABH: Adult Brain Hemisuction
CFA: Caudal Forelimb Area
CST: Corticospinal Tract
CCL: Chemokine (C-C) Ligand
CXCL: Chemokine (C-X-C) Ligand
FG: Fluorogold
GAPDH: Glyceraldehyde 3-Phosphate Dehydrogenase
GEO: Gene Expression Omnibus
IL6: Interleukin 6
IRG1: Immunoresponsive Gene 1
KO: Knockout
lncRNA: Long Noncoding RNAs
NBH: Neonatal Brain Hemisuction
NCBI: National Center for Biotechnology Information
NHS: Normal Horse Serum
PKCγ: Protein Kinase-C gamma
RFA: Rostral Caudal Forelimb
SMC: Sensorimotor Cortex
TTR: transthyretin
XIST: X-Inactive Specific Transcript

## References

[1] Devlin A.M., Cross J.H., Harkness W., Chong W.K., Harding B., Vargha-Khadem F., Neville B.G. (2003). Clinical outcomes of hemispherectomy for epilepsy in childhood and adolescence. Brain.

[2] Peacock W.J., Wehby-Grant M.C., Shields W.D., Shewmon D.A., Chugani H.T., Sankar R., Vinters H.V. (1996). Hemispherectomy for intractable seizures in children: A report of 58 cases. Childs Nerv. Syst..

[3] Van Empelen R., Jennekens-Schinkel A., Buskens E., Helders P.J., van Nieuwenhuizen O. (2004). Functional consequences of hemispherectomy. Brain.

[4] Van Empelen R., Jennekens-Schinkel A., Gorter J.W., Volman M.J., van Nieuwenhuizen O., Helders P.J. (2005). Epilepsy surgery does not harm motor performance of children and adolescents. Brain.

[5] Wilson P.J. (1970). Cerebral hemispherectomy for infantile hemiplegia: A report of 50 cases. Brain.

[6] Anderson V., Spencer-Smith M., Leventer R., Coleman L., Anderson P., Williams J., Greenham M., Jcobs R. (2009). Childhood brain insult: Can age at insult help us predict outcome?. Brain.

[7] Benecke R., Meyer B.U., Freund H.J. (1991). Reorganisation of descending motor pathways in patients after hemispherectomy and severe hemispheric lesions demonstrated by magnetic brain stimulation. Exp. Brain Res..

[8] McClelland S., Maxwell R.E. (2007). Hemispherectomy for intractable epilepsy in adults: The first reported series. Ann. Neurol..

[9] Nemati F., Kolb B. (2012). Recovery from medial prefrontal cortex injury during adolescence: Implications for age-dependent plasticity. Behav. Brain Res..

[10] Yager J.Y., Wright S., Armstrong E.A., Jahraus C.M., Saucier D.M. (2006). The influence of aging on recovery following ischemic brain damage. Behav. Brain Res..

[11] Gianino S., Stein S.A., Li H., Lu X., Biesiada E., Ulas J., Xu X.M. (1999). Postnatal growth of corticospinal axons in the spinal cord of developing mice. Brain Res. Dev. Brain Res..

[12] Gribnau A.A., de Kort E.J., Dederen P.J., Nieuwenhuys R. (1986). On the development of the pyramidal tract in the rat: II. An anterograde tracer study of the outgrowth of the corticospinal fibers. Anat. Embryol. (Berl.).

[13] Joosten E.A., van Eden C.G. (1989). An anterograde tracer study on the development of corticospinal projections from the medial prefrontal cortex in the rat. Brain Res. Dev. Brain Res..

[14] Joosten E.A., Schuitman R.L., Vermelis M.E., Dederen P.J. (1992). Postnatal development of the ipsilateral corticospinal component in rat spinal cord: a light and electron microscopic anterograde HRP study. J. Comp. Neurol..

[15] Gorgels T.G. (1990). A quantitative analysis of axon outgrowth, axon loss, and myelination in the rat pyramidal tract. Brain Res. Dev. Brain Res..

[16] Hsu J.Y., Stein S.A., Xu X.M. (2006). Development of the corticospinal tract in the mouse spinal cord: A quantitative ultrastructural analysis. Brain Res..

[17] Oudega M., Varon S., Hagg T. (1994). Distribution of corticospinal motor neurons in the postnatal rat: Quantitative evidence for massive collateral elimination and modest cell death. J. Comp. Neurol..

[18] Stanfield B.B., O’Leary D.D., Fricks C. (1982). Selective collateral elimination in early postnatal development restricts cortical distribution of rat pyramidal tract neurones. Nature.

[19] Kamiyama T., Yoshioka N., Sakurai M. (2006). Synapse elimination in the corticospinal projection during the early postnatal period. J. Neurophysiol..

[20] Yoshioka N., Murabe N., Sakurai M. (2009). Regressive events in rat corticospinal axons during development in *in vitro* slice cocultures: Retraction, amputation, and degeneration. J. Comp. Neurol..

[21] Canty A.J., Murphy M. (2008). Molecular mechanisms of axon guidance in the developing corticospinal tract. Prog. Neurobiol..

[22] Semeralul M.O., Boutros P.C., Likhodi O., Okey A.B., van Tol H.H., Wong A.H. (2006). Microarray analysis of the developing cortex. J. Neurobiol..

[23] Harel N.Y., Strittmatter S.M. (2006). Can regenerating axons recapitulate developmental guidance during recovery from spinal cord injury?. Nat. Rev. Neurosci..

[24] Takahashi M., Vattanajun A., Umeda T., Isa K., Isa T. (2009). Large-scale reorganization of corticofugal fibers after neonatal hemidecortication for functional restoration of forelimb movements. Eur. J. Neurosci..

[25] Umeda T., Takahashi M., Isa K., Isa T. (2010). Formation of descending pathways mediating cortical command to forelimb motoneurons in neonatally hemidecorticated rats. J. Neurophysiol..

[26] Z’Graggen W.J., Fouad K., Raineteau O., Metz G.A., Schwab M.E., Kartje G.L. (2000). Compensatory sprouting and impulse rerouting after unilateral pyramidal tract lesion in neonatal rats. J. Neurosci..

[27] Yoshikawa A., Atobe Y., Takeda A., Kamiya Y., Takiguchi M., Funakoshi K. (2011). A retrograde tracing study of compensatory corticospinal projections in rats with neonatal hemidecortication. Dev. Neurosci..

[28] Metz G.A., Whishaw I.Q. (2002). Cortical and subcortical lesions impair skilled walking in the ladder rung walking test: A new task to evaluate fore- and hindlimb stepping, placing, and co-ordination. J. Neurosci. Methods.

[29] Starkey M.L., Barritt A.W., Yip P.K., Davies M., Hamers F.P., McMahon S.B., Bradbury E.J. (2005). Assessing behavioural function following a pyramidotomy lesion of the corticospinal tract in adult mice. Exp. Neurol..

[30] Ghosh A., Sydekum E., Haiss F., Peduzzi S., Zörner B., Schneider R., Baltes C., Rudin M., Weber B., Schwab M.E. (2009). Functional and anatomical reorganization of the sensory-motor cortex after incomplete spinal cord injury in adult rats. J. Neurosci..

[31] Tennant K.A., Adkins D.L., Donlan N.A., Asay A.L., Thomas N., Kleim J.A., Jones T.A. (2011). The organization of the forelimb representation of the C57BL/6 mouse motor cortex as defined by intracortical microstimulation and cytoarchitecture. Cereb. Cortex.

[32] Umeda T., Isa T. (2011). Differential contributions of rostral and caudal frontal forelimb areas to compensatory process after neonatal hemidecortication in rats. Eur. J. Neurosci..

[33] Santos S.D., Lambertsen K.L., Clausen B.H., Akinc A., Alvarez R., Finsen B., Saraiva M.J. (2010). CSF transthyretin neuroprotection in a mouse model of brain ischemia. J. Neurochem..

[34] Lin H.Y., Davis F.B., Luidens M.K., Mousa S.A., Cao J.H., Zhou M., Davis P.J. (2011). Molecular basis for certain neuroprotective effects of thyroid hormone. Front. Mol. Neurosci..

[35] Brown C.J., Ballabio A., Rupert J.L., Lafreniere R.G., Grompe M., Tonlorenzi R., Willard H.F. (1991). A gene from the region of the human X inactivation centre is expressed exclusively from the inactive X chromosome. Nature.

[36] Ueno M., Hayano Y., Nakagawa H., Yamashita T. (2012). Intraspinal rewiring of the corticospinal tract requires target-derived brain-derived neurotrophic factor and compensates lost function after brain injury. Brain.

[37] Liu Z., Zhang R.L., Li Y., Cui Y., Chopp M. (2009). Remodeling of the corticospinal innervation and spontaneous behavioral recovery after ischemic stroke in adult mice. Stroke.

[38] Nishibe M., Barbay S., Guggenmos D., Nudo R.J. (2010). Reorganization of motor cortex after controlled cortical impact in rats and implications for functional recovery. J. Neurotrauma.

[39] Michelucci A., Cordes T., Ghelfi J., Pailot A., Reiling N., Goldmann O., Binz T., Wegner A., Tallam A., Rausell A. (2013). Immune-responsive gene 1 proteins links metabolism to immunity by catalyzing itaconic acid production. Proc. Natl. Acad. Sci. USA.

[40] Wu P., Zuo X., Deng H., Liu X., Liu L., Ji A. (2013). Roles of long noncoding RNAs in brain development, functional diversification and neurodegenerative diseases. Brain Res. Bull..

[41] Qureshi I.A., Mehler M.F. (2010). Genetic and epigenetic underpinnings of sex differences in the brain and in neurological and psychiatric disease susceptibility. Prog. Brain Res..

[42] Qureshi I.A., Mehler M.F. (2010). The emerging role of epigenetics in stroke: II. RNA regulatory circuitry. Arch. Neurol..

[43] Pronichev I.V., Lenkov D.N. (1998). Functional mapping of the motor cortex of the white mouse by a microstimulation method. Neurosci. Behav. Physiol..

[44] Paxonos G., Franklin K.B.J. (1996). The Mouse Bain in Sterotaxic Coordinates.

[45] Hori M., Nakamachi T., Rakwal R., Shibato J., Nakamura K., Wada Y. (2012). Unraveling the ischemic brain transcriptome in a permanent middle cerebral artery occlusion mouse model by DNA microarray analysis. Dis. Model Mech..

[46] Hori M., Nakamachi T., Rakwal R., Shibato J., Ogawa T., Aiuchi T., Tsuruyama T., Tamaki K., Shioda S. (2012). Transcriptomics and proteomics analyses of the PACAP38 influenced ischemic brain in permanent middle cerebral artery occlusion model mice. J. Neuroinflamm..

[47] Ogawa T., Rakwal R., Shibato J., Sawa C., Saito T., Murayama A., Kuwagata M., Kageyama H., Yagi M., Satoh K. (2011). Seeking gene candidates responsible for developmental origins of health and disease. Congenit. Anom. (Kyoto).

[48] Ogawa T., Shibato J., Rakwal R., Saito T., Tamura G., Kuwagata M., Shioda S. (2014). Seeking genes responsible for developmental origins of health and disease from the fetal mouse liver following maternal food restriction. Congenit. Anom. (Kyoto).

